# Scrolling to apathy? The relationship between short video addiction and the “lying flat” tendency among Chinese university students

**DOI:** 10.3389/fpsyg.2026.1685559

**Published:** 2026-02-27

**Authors:** Jie Xu, Adibah Ismail, Thurasamy Ramayah, Christine Wan-Shean Liew, Chuan Zheng

**Affiliations:** 1 School of Literature and Media, Chaohu University, Hefei, China; 2 School of Multimedia Technology and Communication, Universiti Utara Malaysia, Alor Setar, Malaysia; 3 School of Management, Universiti Sains Malaysia, Minden Heights, Penang, Malaysia; 4 Business School, Monash University Malaysia, Bandar Sunway, Selangor, Malaysia; 5 School of Business Management, College of Business, Universiti Utara Malaysia, Sintok, Malaysia; 6 Luzhou Vocational and Technical College, Luzhou, Sichuan, China; 7 Sunway Business School (SBS), Sunway University, Petaling Jaya, Selangor, Malaysia; 8 Department of Information Technology & Management, Daffodil International University, Birulia, Bangladesh; 9 Faculty of Business, Sohar University, Sohar, Oman; 10 Asia Pacific University of Technology & Innovation (APU), Kuala Lumpur, Malaysia; 11 University Center for Research & Development (UCRD), Chandigarh University, Ludhiana, Punjab, India; 12 School of Business, The University of Jordan (UJ), Amman, Jordan

**Keywords:** flow experience, lying flat phenomenon, peer influences, platform attachment, short video addiction

## Abstract

**Background:**

The increasing prevalence of short video platforms among Chinese university students has raised concerns about their potential threat to psychological well-being and lifestyle choices, particularly the emergence of the “lying flat” phenomenon. While existing studies have explained the “lying flat” phenomenon from cultural and social perspectives, few have examined its interaction with specific media use behaviors, particularly within the immersive and addictive environment of short video platforms.

**Objective:**

Based on the social cognitive theory, this study investigated the psychological mechanisms underlying this phenomenon, focusing on the mediating role of short video addiction in the relationship between flow experience, platform attachment, and peer influence, as well as the moderating role of self-efficacy.

**Methods:**

An online survey was administered to Chinese university students, yielding 486 valid responses. Data were analyzed using structural equation modeling (SEM).

**Results:**

The results indicate that higher levels of short video addiction are associated with a greater tendency to adopt a “lying flat” attitude. Flow experience, platform attachment, and peer influence contributed to increased short video addiction, which mediated their indirect effects on the “lying flat” tendencies. However, the interaction effect between self-efficacy and SV addiction does not significantly predict lying flat tendency.

**Conclusion:**

These findings provide new insights into how digital media use influences youth lifestyle disengagement and offer practical implications for managing SV addiction among university students.

## 1 Introduction

As of 2025, the global number of internet users has exceeded 6 billion (International Telecommunication Union, 2025). Short video (SV) platforms, characterized by their smartphone-based accessibility, quick editing features, and ease of sharing, have emerged as a significant global phenomenon. Research indicates that SVs have not only reshaped media consumption habits but also exerted a profound impact on users’ attitudes and behaviors. For instance, the study by Xu et al. (2024) demonstrated the rapid changes in SV content trends and the high level of public engagement. Additionally, Ye et al. (2022) found that increased engagement with SVs predicts stronger user dependence, whereas SV addiction impairs intrinsic and extrinsic learning motivation. These studies indicate that SVs have changed media consumption habits and possess a unique influence. In China, the penetration of SV platforms is particularly significant. By 2025, the number of internet users in the country had reached 1.123 billion, of which 1.068 billion were SV users, accounting for 95.1% of the total (China Internet Network Information Center, 2025). Platforms such as Douyin and Kuaishou have evolved beyond their role as primary entertainment sources to become integral elements of China’s social and cultural landscape.

University students, as digital natives of Generation Z, are proficient in using digital technologies and actively rely on platforms like TikTok and Instagram for social interaction and information acquisition. They tend to prefer short-form, visually engaging content and seek immediate gratification, which may increase their susceptibility to excessive media consumption. Moreover, engaging and immersive SV experiences may raise the risk of overuse, potentially leading to addictive behaviors (Huang et al., 2022). Such addiction has been shown to impair students’ learning motivation and academic performance and further disrupt their social relationships and mental health (Xiao et al., 2024; Ye et al., 2025a; Ye et al., 2025b).

The concept of “lying flat” has emerged in recent years as a social phenomenon in China. It refers to a deliberate choice to abandon the pressures and pursuits associated with social competition and consumerism, instead opting for a simpler, low-pressure lifestyle (Zheng et al., 2023). This phenomenon primarily reflects the frustration and protest of some young people against the intense societal competition and the perceived disproportionate rewards for personal effort (Hsu, 2022). “Lying flat” challenges and redefines traditional notions of success, which may lead to long-term shifts in social values and expectations.

When university students become addicted to SVs, it can lead to distraction, reduced focus, and decreased learning efficiency (Ye et al., 2023a; Ye et al., 2023b). Existing studies have shown that SV addiction significantly undermines both intrinsic and extrinsic learning motivation and decreases students’ sense of learning well-being (Ye et al., 2022; Zhan and Zhu, 2025; Sun et al., 2023). The decline in motivation and well-being further contributes to emotional exhaustion and psychological disengagement (Güngör and Sari, 2022). Such exhaustion often prompts individuals to adopt avoidance and withdrawal as psychological defense strategies, resulting in “energy-retreat” behaviors. These passive coping tendencies are psychologically consistent with the characteristics of the “lying flat” mentality (Montero-Marin et al., 2014; Qi, 2021). In addition, research indicates that students with lower self-control and weaker future time orientation are more susceptible to SV addiction, tending toward instant gratification and abandoning long-term goals (Liu et al., 2025). Prolonged immersion in the instant entertainment offered by SVs can weaken the ability to delay gratification and increase feelings of fatigue and helplessness in real life. Meanwhile, SV addiction has also been found to be closely associated with loneliness and negative cognitive bias, which may reduce students’ interest in real life and social participation, thereby fostering avoidance and passive coping behaviors (Zhao and Kou, 2024). While extensive studies have examined the psychological and motivational outcomes of SV addiction, limited attention has been paid to its broader behavioral implications, particularly its association with the emerging “lying flat” phenomenon. Therefore, the relationship between SV addiction and the phenomenon of “lying flat” among university students requires further investigation. Empirical research is needed to obtain concrete data to explore the influencing factors and mechanisms behind this behavior.

However, existing research has primarily explained the “lying flat” phenomenon from cultural and sociological perspectives. Some scholars have argued that “lying flat” reflects a form of emotional adjustment among young people as they navigate prevailing social norms and expectations, carrying profound historical and affective symbolism (Zhang and Li, 2023). Su (2023) further suggests that the “lying flat” attitude, together with the “sang culture” and “diaosi culture,” represents a form of symbolic expression through which young people articulate their experiences of life stresses. Meanwhile, several scholars have begun to approach the phenomenon from a psychological perspective, exploring the relationship between “lying flat,” individual mental health, and behavioral patterns. For instance, Lu et al. (2024) found that a stronger tendency toward “lying flat” undermines college students’ mental health and academic engagement, while Zhu et al. (2025) revealed that “lying flat” indirectly triggers excessive smartphone use by lowering self-esteem. Although these studies have enriched the understanding of the “lying flat” phenomenon, few have systematically examined its underlying psychological mechanisms in relation to specific media use behaviors. In particular, within the highly immersive and potentially addictive environment of SV platforms, the ways in which individuals’ psychological and social experiences contribute to “lying flat” tendencies through the mediating role of SV addiction remain insufficiently explored from an empirical perspective. Furthermore, while existing studies have begun to address psychological factors such as self-esteem and self-efficacy, the moderating role of self-efficacy in the relationship between SV addiction and “lying flat” behavior remains underexplored.

The Social Cognitive Theory (SCT) emphasizes that individual behavior results from the dynamic interaction among external environmental factors, internal psychological states, and personal determinants (Bandura, 1986). This framework provides a robust theoretical foundation for the present study. On one hand, university students are influenced by social and environmental stimuli such as peer effects, flow experiences, and platform attachment during immersive media use. These external factors shape individuals’ cognitive and emotional experiences, which may trigger SV addiction (Ye et al., 2025a; Ye et al., 2025b). On the other hand, such addictive behaviors not only reflect excessive reliance on technology use but may also deplete individuals’ psychological resources (Ye et al., 2024a; Ye et al., 2024b), potentially manifesting as a “lying flat” tendency, which denotes an avoidant and passive coping style. Meanwhile, self-efficacy, a central construct within SCT, determines whether individuals possess sufficient self-regulatory capacity to buffer environmental pressures and behavioral impulses, thereby moderating the relationship between addiction and the “lying flat” tendency (Wachs et al., 2020). Therefore, SCT not only elucidates how external social environments influence university students’ media addiction through internal psychological mechanisms but also reveals how these behaviors further shape disengaged coping tendencies. This theoretical perspective thus provides a comprehensive framework for constructing the integrative pathway model proposed in this study.

The current research aims to examine the determinants and possible mechanisms of “lying flat” tendency among the university students, focusing on the mediating role of SV addiction in the relationship between flow experience, platform attachment, peer influences, and “lying flat,” as well as the moderating effect of self-efficacy on the link between SV addiction and “lying flat” phenomenon. Using a quantitative research approach, the study will collect data through a survey and analyze the relationships between these variables using structural equation modeling (SEM). This research will deepen the understanding of how SV use behavior among university students affects their attitudes towards life, particularly how flow experience, platform attachment, and peer influences shape their life and study choices. This research framework provides new insights into how SV addiction relates to the development of ‘lying flat’ mindset among university students, offering strategies for educators and policymakers on how to effectively manage and guide the healthy use of SVs among university students.

## 2 Literature review and hypothesis development

### 2.1 Social cognitive theory

Social cognitive theory (SCT), proposed by Albert Bandura, provides a critical theoretical framework for understanding and predicting individual attitude or behavior in this study (Bandura, 2023). It particularly emphasizes the role of self-efficacy and the interaction between individuals and their environment in driving behavioral changes. SCT highlights mechanisms such as observational learning, modeling, reinforcement, and reciprocal determinism, emphasizing that behavior is shaped not only by internal cognition but also by social and environmental interactions. It has been widely adopted in studies examining individual behavior in digital environments, particularly among young populations (Sokolova et al., 2024). Given its emphasis on the interaction between individual agency and environmental factors, SCT is particularly well-suited to explain how university students develop behavioral tendencies, such as “lying flat” in increasingly digital and socially mediated environments. Its core principles offer a valuable lens through which to understand the social learning, self-efficacy, and peer influence mechanisms that shape students’ behavioral responses in such contexts.

### 2.2 SV flow

The concept of flow was originally proposed by Csikszentmihalyi (1990) to describe a state of deep immersion and enjoyment that arises when individuals engage in goal-directed tasks requiring focused attention and a balance between challenge and skill. While SV viewing does not meet these classical conditions, research suggest that its algorithm-driven, immersive design can induce similar psychological states, known as SV flow (Huang et al., 2022). SV flow is thus defined as a positive experience characterized by focused attention, enjoyment, and a distorted sense of time while consuming SV content (Huang et al., 2022). With the growing popularity of short video platforms, scholars have conducted extensive research on SV flow. For example, Zhao and Wagner (2022) highlighted the role of recommendation algorithms and multimodal interactions in promoting flow states and increasing users’ attachment to the platform. Additionally, Huang et al. (2021) confirmed that while flow experiences enhance enjoyment, prolonged use may lead to problematic behaviors such as dependency and cognitive fatigue. Furthermore, Gan (2024) revealed that flow experience positively impacts user satisfaction by reducing regret and fostering a sense of accomplishment. These studies provide a conceptual framework for understanding flow experience in SV viewing and its role in user behavior, highlighting its key role in enhancing user engagement and satisfaction. However, it is important to note that flow experience in SV use can also have negative consequences, such as problematic use and cognitive overload.

For university student users, SV flow experience may lead to excessive immersion, resulting in time management issues. When engagement in SVs replaces essential real-life activities such as study or social interaction, it may result in psychological strain over time. This study focuses on the long-term effects of flow experience on university students’ mental health. When exploring the psychological motivations behind university students’ choice to “lie flat,” SV flow experience may play a crucial role. Intense flow experiences might lead individuals to seek a sense of achievement and satisfaction in the virtual world rather than pursuing professional and academic goals in real life.

### 2.3 SV addiction

In this study, short video addiction is defined as a form of behavioral addiction that involves non-substance psychological dependence and is characterized by compulsive, uncontrolled use of short video platforms that interferes with daily functioning (Beard and Wolf, 2001). Although distinct from clinical substance addiction, it presents key behavioral symptoms such as loss of control, withdrawal, and tolerance (Block, 2008). These symptoms align with the dimensions commonly evaluated in established instruments for behavioral addiction or problematic media use, such as the Chen Internet Addiction Scale, which assesses compulsive use, withdrawal, and tolerance (Chen et al., 2003). Zhang et al. (2019) found that social interaction anxiety and social isolation are positively correlated with interpersonal attachment, while personalization and entertainment are positively correlated with site attachment, both of which contribute to the formation of SV addiction. Additionally, Lu et al. (2022) conducted an empirical study on SV addiction among adolescents, indicating that boredom in daily life and the perception of user-generated content (UGC) significantly influence the level of SV addiction. These studies provide a comprehensive perspective on SV addiction, emphasizing the role of individual characteristics and social environmental factors in the formation of SV addiction.

The flow experience of university students while watching SVs may contribute to their addiction to SVs. Flow experience can drive users to engage with SV applications more frequently, thereby increasing the risk of addiction. For example, Lu et al. (2022) emphasized how flow experience increases the risk of addiction among adolescents by enhancing users’ dependence on SV applications. Therefore, the higher the flow experience university students have with SVs, the more likely they are to develop SV addiction. Based on this, the study proposes the following research hypothesis:

*H1*: The flow experience of university students with SVs is positively associated with their SV addiction.

### 2.4 Platform attachment

Platform attachment refers to the emotional connection and dependency that users develop when using social media or other online platforms. This attachment usually involves users’ satisfaction, trust, and emotional ties to the platform, making it a crucial factor influencing users’ continued use of the platform (Yang et al., 2021). Short-video platform attachment specifically refers to users’ deep dependency and emotional connection with SV platforms, which may stem from the platform’s interactivity and entertainment value (Zhang et al., 2019). Users may develop this attachment due to the platform’s social functions, the attractiveness of its content, or the need for personal expression. The significance of platform attachment lies in its ability to significantly influence users’ behavior patterns, such as increasing use frequency and enhancing user interaction with platform content. The study by Yang et al. (2021) indicated that users’ sense of co-created value through social media leads to changes in psychological needs and opportunities for self-expression, which mediate the formation of attachment between users and social media. Dwivedi et al. (2019) found that emotional attachment, by enhancing brand credibility and user satisfaction, significantly affects users’ brand-related equity.

Platform attachment involves the emotional connection and dependency that users develop with a specific SV platform. This dependency not only increases user engagement but may also lead to excessive use and addictive behavior. For example, Zhang et al. (2019) analyzed the factors contributing to SV application addiction from the perspective of socio-technical and attachment theories, finding that social interaction anxiety and social isolation are closely linked to individuals’ attachment to SV platforms, thereby influencing addictive behavior. Similarly, Liu and Xie (2025) found that emotional attachment to short-form video platforms is positively associated with SV addiction among college students. These studies suggest that platform attachment may increase the likelihood of addiction by deepening users’ emotional investment in SV content and social connections. Therefore, when university students’ attachment to a specific platform increases, it may lead to SV addiction. Based on this, this study proposes the following research hypothesis:

*H2*: The degree of platform attachment among university students is positively associated with their SV addiction.

### 2.5 Peer influence

Peer influence refers to the phenomenon where an individual’s behavior or attitudes are affected by the surrounding peer group. Peer groups play an important role in the socialization process of individuals, especially during adolescence and university years. Peer influence has been widely studied in sociology and psychology, particularly in the context of education, workplaces, and social networks. In educational settings, peer influence can affect students’ learning motivation and academic performance. For example, Poldin et al. (2016) found that social connections established with study partners have a positive peer effect on students’ academic performance. In the workplace, peer influence may be related to job satisfaction and organizational commitment, which can impact employees’ work performance and career development (Cornelissen et al., 2017). Additionally, the mechanisms of peer influence may vary across different cultures and social structures. In schools, peer influence is typically related to academic achievement and social behavior (Poldin et al., 2016; Ye et al., 2024a; Ye et al., 2024b). Furthermore, research has found that peer influence on social media platforms significantly impacts personal identity and behavior patterns (Ye et al., 2022).

Peer influence among university students is positively associated with SV addiction. Peer influence involves the effect that peers have on an individual’s behavior which is especially pronounced in the use of digital media. For example, the study by Ye et al. (2022) found that the flow experience of SVs has a positive effect on SV addiction, and interactions and sharing behaviors among peers may further strengthen this effect. Additionally, the study by Kang et al. (2023) highlighted the moderating role of peer communication within the family environment, suggesting that peer influence is a critical factor in shaping adolescent SV addiction by either mitigating or exacerbating addictive behaviors. These studies underscore the pivotal role of peer interactions in driving SV addiction. Based on this, the study proposes the following research hypothesis H3:

*H3*: The degree of peer influence among university students is positively associated with their SV addiction.

### 2.6 The “lying flat” mentality

“Lying flat” is a social and cultural phenomenon, particularly popular among young people in China, that refers to a deliberate choice to abandon traditional societal goals and the pressures of striving, in favor of a simpler and lower-pressure lifestyle. This phenomenon reflects the younger generation’s response to the intense work and life pressures, as well as their rejection of the ever-increasing societal expectations (Hsu, 2022). For example, Zheng et al. (2023) explored how personal relative deprivation can lead to a diminished motivation for self-improvement, which may, in turn, cause individuals to choose the “lying flat” lifestyle. Social comparison and perceived injustice are used to explain why some university students and young people opt for this way of life. The emergence of the “lying flat” mentality is a critique of the pressures of contemporary work and life expectations, highlighting the imbalance between work and life in modern society and the neglect of individual well-being. This phenomenon reveals a sense of frustration and resistance among some young people toward the future, prompting reflection and challenges to societal, educational, and economic policies. “Lying flat” is not just a change in individual behavior, but also a questioning of the current economic development model and social values (Zhou, 2023).

University students’ SV addiction may be positively related to their choice to “lie flat.” SV addiction can lead students to be more psychologically and behaviorally inclined to escape the pressures and challenges of real life, choosing a more passive attitude and behavior. For instance, Ye et al. (2022) found that SV addiction significantly reduces both intrinsic and extrinsic learning motivation, negatively impacting students’ learning well-being. This negative impact may encourage students to adopt a “lying flat” tendency as a response to the lack of motivation for learning and social participation. Ye et al. (2023a) and Ye et al. (2023b) further demonstrated that SV addiction is significantly positively correlated with learning avoidance motivation, indicating that higher levels of SV addiction are associated with stronger tendencies to avoid academic responsibilities and tasks. Additionally, Liu et al. (2021) showed that there is a positive correlation between SV addiction and perceived stress, which may further contribute to avoidant responses. As suggested by Qi (2021), such avoidance and passivity reflect a psychological escapism that characterizes the “lying flat” mentality. These studies suggest that SV addiction may lead to psychological and behavioral changes in university students, prompting them to choose “lying flat” as a way to cope with the excessive consumption of media and societal pressure. Based on this, the study proposes the following research hypothesis:

*H4*: The degree of SV addiction among university students is positively related to their “lying flat” mentality.

Short video flow provides users with intense psychological satisfaction during SV viewing, which encourages repeated engagement and may eventually lead to addictive use patterns (Qin et al., 2022). Research has shown that SV addiction is significantly negatively correlated with university students’ social participation. Those addicted to SVs often exhibit lower levels of social participation and higher levels of isolation (Huang et al., 2021). This sense of isolation and low participation may makes them more likely to choose to “lie flat,” meaning they abandon active participation in social competition and effort. In addition, Nong et al. (2023) demonstrated that SV flow indirectly reduces students’ achievement motivation through SV addiction, highlighting the mediating role of addiction in the relationship between immersive experiences and real-life goal pursuit. Similarly, Ye et al. (2022) found that SV immersion not only fosters addiction, but that such addiction further diminishes students’ learning well-being in real life. These findings suggest that SV addiction functions as a critical psychological bridge linking SV flow and the tendency to “lie flat.” When university students become immersed in the instant gratification offered by SV platforms, they may gradually develop addictive patterns of use. Such addictive behaviors further undermine their motivation and engagement with real-life goals, thereby increasing the likelihood of adopting a “lying flat” attitude. Thus, this study formulates the following hypothesis.

*H5*: The flow experience of university students with SVs is positively associated with their tendency to “lie flat” through SV addiction.

Short-video platform attachment refers to the high dependency and emotional connection that users develop with SV platforms, which leads to frequent use and eventually results in addiction (Zhang et al., 2019). This addictive behavior may further influences the lifestyle and behavioral choices of university students. A study on Chinese adolescents found that SV use, by fulfilling individual needs, triggers addictive behavior, which in turn is indirectly associated with individual depression levels, highlighting the mediating role of SV addiction between platform use motivation and psychological states (Zhu et al., 2025). Additionally, in the context of social media, research has shown that social media addiction, through internet addiction and “phubbing” behavior, is indirectly associated with individuals’ depression, anxiety, and stress levels, further emphasizing addiction as a psychological bridge between media use and issues related to real-life adaptation (Ergün et al., 2025). Therefore, SV addiction is not only a result of platform attachment but may also weaken university students’ ability to cope with real-life challenges and pursue goals, potentially giving rise to the “lying flat” behavior. Building on the above analysis, this study posits the following hypothesis:

*H6*: The attachment of university students to short-video platforms is positively related to their tendency to “lie flat” through SV addiction.

University students are influenced by their peers within peer groups, including in their use and attachment to short-video platforms (Lu et al., 2022). When peers frequently engage with short-video platforms, individuals become more susceptible to influence, increasing their use frequency and attachment to these platforms, ultimately culminating in addictive behavior. Peer influence amplifies the effects of SV addiction through mechanisms such as imitation and social comparison. In addition, prior research has shown that SV addiction is not only a result of social environmental influence, but may also act as a bridge between external factors and individual psychological or behavioral responses. For example, Mu et al. (2022) found that SV addiction significantly mediated the relationship between social anxiety and negative psychological outcomes among adolescents. Zhu et al., (2025) further revealed that SV use influenced depression through gratification and addictive tendencies, suggesting that addiction serves as a critical mediating mechanism linking external stimuli to emotional responses. For university students, the imitative drive and immediate gratification induced by peers’ frequent SV use may gradually foster dependency. As real-world pressures accumulate, such dependency may provide a pathway for emotional escape, eventually manifesting in a “lying flat” tendency as a passive coping strategy. Therefore, this study proposes the following research hypothesis:

*H7*: Peer influence among university students is positively associated with their tendency to “lie flat” through SV addiction.

### 2.7 Self-efficacy

In Social Cognitive Theory, self-efficacy refers to an individual’s belief and confidence in their ability to successfully complete specific tasks or handle particular situations. It plays a crucial role in many psychological behavior patterns (Bandura, 1986). For university students, self-efficacy not only affects their academic achievements but also positively related to their behavioral choices and mental health (Cao et al., 2020). University students with high self-efficacy are more likely to set and pursue positive goals and take effective actions to achieve these goals. They are better able to manage their time and resources, cope with challenges in academics and life, and thus improve their overall quality of life and well-being (Freire et al., 2020).

Self-efficacy can help university students more effectively manage their time and control their use of SVs, thereby reducing addictive behaviors (Lin et al., 2023). Additionally, Zhou et al. (2022) pointed out that the higher self-efficacy, the better students can resist the pressure from addictive behaviors, thereby reducing the occurrence of academic burnout. Peng et al. (2022) studied anxiety, addiction, and subjective well-being among SV users. Their results showed that SV addiction decreases users’ subjective well-being, but individuals with high self-efficacy are better able to cope with these negative emotions, maintaining higher life satisfaction and positive behaviors. These studies collectively indicate that self-efficacy acts as a buffer between SV addiction and “lying flat” tendency, significantly weakening the association between them. Therefore, this study formulates the following research hypothesis:

*H8*: University students’ self-efficacy weakens the positive relationship between SV addiction and “lying flat” tendency.

**Figure 1:**
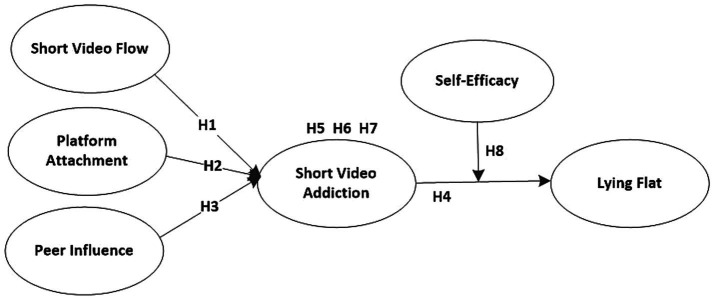
Research conceptual model.

Based on the research hypotheses, the current investigation proposes the research model shown in Figure 1.

## 3 Method

### 3.1 Participants and data collection

The primary objective of this study is to examine the relationships among short video flow, peer influence, platform attachment, short video addiction, and the “lying flat” tendency among Chinese university students. The research subjects are therefore Chinese university students. According to statistics from the Ministry of Education of the People’s Republic of China, there are 3,074 universities in China, with a total student population of 47.63 million as of 2023 (Ministry of Education of the People’s Republic of China, 2024). Moreover, a survey conducted by China Youth Network on SV use among university students revealed that 30% of university students spend 2–5 h per day on SV platforms (Sina Education, 2022).

Data were collected using “Wenjuanxing,” a professional Chinese online survey platform with over 4 million registered users. Due to the substantial size of the university student population and the absence of a complete sampling frame, a convenience sampling method was employed. Screening questions were included to ensure that respondents were current university students and regular users of SV platforms, with questions on their daily usage frequency and duration. As all items in the online questionnaire were set as mandatory within the platform, no missing data was observed. The survey was conducted from December1 to December 30, 2024, and yielded 486 valid responses. A power analysis using G*Power 3.1 indicated that a minimum sample size of 119 was required. Therefore, the final sample of 486 respondents was considered sufficient for the statistical analyses. Participation was entirely voluntary and anonymous. Before completing the questionnaire, participants were informed of the purpose of the study, their right to withdraw at any time, and the confidential handling of their responses. Moreover, according to the institutional guidelines and local regulations, this study was exempted from formal ethical review, as it involved non-intrusive and anonymous data collection with no identifiable personal information. The exemption was confirmed by the relevant ethics committee at the authors’ institution.

### 3.2 Measurement tools

The independent variables include SV flow, platform attachment, and peer influence, with “SV addiction” as the mediating variable and “lying flat” as the dependent variable. Self-efficacy serves as the moderating variable. The measurement tools are divided into two parts: first, the demographic variables of the sample, which include basic information such as gender, major, and grade level. Second, in terms of research variables, this study uses a seven-point Likert scale, where 1 indicates that the sample strongly disagrees with the item content, and 7 indicates strong agreement. The design of the research variable items is based on established and validated scales (see Appendix).

#### 3.2.1 SV flow

The operational definition of university students’ SV flow is measured by their flow experience while watching SVs, which includes their complete immersion in the video content, altered sense of time, sense of control, perceived enjoyment, and degree of deep focus. This study references the work of Hong et al. (2016), which examined how Internet cognitive failure affects gamers’ cognitive anxiety and flow experience. The flow experience dimension in their study reported CR = 0.94 and *α* = 0.94. In the current study, the Cronbach’s alpha for this scale was 0.849, indicating good internal reliability. The item content was modified to fit the context of this research, resulting in four items. For example, “When I watch SVs, I am completely immersed in the content and forget my surroundings.”

#### 3.2.2 Platform attachment

The operational definition of SV platform attachment is measured by assessing users’ emotional attachment to the platform, use frequency, sense of dependence on the platform, viewing the platform as an indispensable part of daily life, and the tendency to recommend the platform to others. This study references the research by Huangfu et al. (2022), which investigated how emotional attachment is significantly influenced by psychological needs and plays a positive role in relationship quality. In their study, the emotional attachment dimension demonstrated good composite reliability (CR = 0.836). In the present study, the Cronbach’s alpha for this scale was 0.921, indicating excellent internal consistency. The items were modified to fit the context of this research, resulting in five items. For example, “I feel somewhat uneasy when I am unable to use the SV platform.”

#### 3.2.3 Peer influence

The operational definition of peer influence in watching SVs is measured by assessing university students’ resonance with their classmates’ opinions about the entertainment value of SVs, the degree to which they value their classmates’ perspectives, the influence of group behavior among classmates, and the sharing of SVs within the peer group. This study references the research by Khlaisang et al. (2023), which assessed the acceptance of using new technology in teaching, where the peer influence dimension consisted of four items, with CR = 0.918. This scale demonstrated good internal reliability in this study, with a Cronbach’s alpha of 0.852. The item content was modified to fit the context of this research, resulting in four items. For example, “My classmates’ opinions about SVs are important to me.”

#### 3.2.4 SV addiction

SV addiction is operationally defined as the excessive consumption of SVs despite the presence of other important tasks. It is characterized by feelings of anxiety or unease when unable to watch, repeated failures to reduce viewing time, and a preference for watching SVs over spending time with friends and family. This study is based on the Chinese Internet Addiction Scale developed by Chen et al. (2003) and its psychometric research, which validated its application in Chinese university student populations. The scale provides a multidimensional assessment of addictive behaviors, including excessive use, functional impairment, emotional dependence, lack of self-control, and social influence. The item content was modified to fit the context of this research, resulting in four items. For example, “I spend a lot of time watching SVs, even when I have other important things to do.” In the present study, this scale demonstrated good internal consistency, with a Cronbach’s alpha of 0.862.

#### 3.2.5 “Lying flat”

In this study, the operational definition of “lying flat” is measured by assessing individuals’ lack of goals and pursuits in life and study, a pessimistic attitude towards personal efforts to change their situation, lack of effort in various aspects, resistance to activities that require effort, a perfunctory approach to tasks, satisfaction with the current state, and avoidance of competition. This study references the work of Lu et al. (2023), which developed six items to assess the tendency towards “lying flat” and conducted cross-validation of the reliability and validity across different samples in China (Cronbach’s *α* = 0.785). In our sample, the scale demonstrated excellent internal consistency, with a Cronbach’s alpha of 0.912. This study adopts the six items from that research. For example, “I have no goals or pursuits in life and study.”

#### 3.2.6 Self-efficacy

The operational definition of self-efficacy refers to an individual’s ability to remain calm, confident, and effectively solve problems when faced with unexpected issues, difficulties, and complex tasks, as well as their confidence and ability to achieve set goals. To design the Likert scale items for the university students’ self-efficacy dimension, this study references the General Self-Efficacy Scale (GSES) developed by Schwarzer (1995), a classic scale frequently used to measure individuals’ sense of self-efficacy in various contexts. This study selected five items and adapted them to fit the context, such as “I can effectively solve unexpected problems”. The scale yielded a Cronbach’s alpha of 0.880, suggesting reliable measurement.

### 3.3 Data analysis

This study utilizes SPSS 26.0 for descriptive statistical analysis to present the frequency and percentage of demographic variables in the sample. Subsequently, Structural Equation Modeling (SEM) was employed via SmartPLS 4.0 to examine the hypothesized relationships among latent variables. Following a two-stage analytical procedure, the measurement model was first evaluated in terms of indicator loadings, internal consistency (Cronbach’s alpha, composite reliability), convergent validity (average variance extracted, AVE), and discriminant validity using the HTMT ratios. The structural model was then assessed based on the significance of path coefficients, *R*^2^ values, effect sizes (f^2^), and predictive relevance, with significance determined via bootstrapping.

## 4 Results

### 4.1 Analysis of demographic information

This study collected a total of 486 valid questionnaires. The majority of respondents were male, with 245 participants, accounting for 50.4%. The predominant field of study was agricultural sciences, with 122 participants (25.1%). Most respondents were first-year students, with 129 participants (26.5%). The statistical results are shown in Table 1 below.

**Table 1:** Analysis of demographic information.

Category	Group	Frequency	Percentage (%)
Gender	Male	245	50.4%
	Female	241	49.6%
Major	Natural sciences	95	19.5%
	Agricultural sciences	122	25.1%
	Medical sciences	98	20.2%
	Engineering and technology sciences	103	21.2%
	Humanities and social sciences	68	14.0%
Year Level	First year	129	26.5%
	Second year	121	24.9%
	Third year	126	25.9%
	Fourth year	110	22.6%

### 4.2 Assessment of measurement model

Since the data was collected from a single source, it is crucial to address the potential issue of Common Method Bias. Following Kock’s (2015) guidelines, a full collinearity test was conducted. The results indicated that all Variance Inflation Factor (VIF) values were below the threshold of 5.0, specifically: SVF = 2.338, PA = 2.423, PI = 1.835, SVA = 3.027, SE = 2.318, and LF = 3.302. These findings confirm that CMB is not a concern in our data.

In line with the recommendations of Hair et al. (2022), we assessed the reliability and validity of the instruments used in our measurement model. As shown in Table 2, the factor loadings for each dimension ranged from 0.774 to 0.903, all exceeding the threshold of 0.7. The Cronbach’s *α* coefficients for the dimensions ranged from 0.849 to 0.921, significantly higher than the critical value of 0.7. The composite reliability values ranged from 0.857 to 0.924, indicating good internal consistency across all dimensions. The AVE values ranged from 0.675 to 0.761, all surpassing the 0.5 standard. Based on these results, the measurement model in this study demonstrates convergent validity.

**Table 2:** Convergent validity analysis.

Constructs	Items	Factor loading	Cronbach’s alpha	Composite reliability	Average variance extracted (AVE)
Short video flow	SVF1	0.862	0.849	0.857	0.688
	SVF2	0.862			
	SVF3	0.797			
	SVF4	0.795			
Platform attachment	PA1	0.853	0.921	0.924	0.761
	PA2	0.900			
	PA3	0.864			
	PA4	0.903			
	PA5	0.839			
Peer influence	PI1	0.774	0.852	0.875	0.691
	PI2	0.829			
	PI3	0.878			
	PI4	0.842			
Short video addiction	SVA1	0.824	0.862	0.867	0.707
	SVA2	0.838			
	SVA3	0.861			
	SVA4	0.841			
Lying flat	LF1	0.818	0.912	0.914	0.695
	LF2	0.862			
	LF3	0.826			
	LF4	0.866			
	LF5	0.779			
	LF6	0.848			
Self-efficacy	SE1	0.857	0.880	0.904	0.675
	SE2	0.828			
	SE3	0.824			
	SE4	0.863			
	SE5	0.730			

SVF = Short Video Flow, PA = Platform Attachment, PI = Peer Influence, SVA = Short Video Addiction, LF = Lying Flat, SE = Self-Efficacy.

Next, discriminant validity is evaluated using the HTMT method. As presented in Table 3, the Heterotrait–Monotrait (HTMT) value are all lower than 0.85 (Hair et al., 2022). This finding confirms that the respondents understood the 6 constructs are distinct. Consequently, the measurement model satisfies all established criteria.

**Table 3:** HTMT criterion.

Variable	LF	PA	PI	SE	SVA	SVF
Lying flat (LF)						
Platform attachment (PA)	0.771					
Peer influence (PI)	0.704	0.578				
Self-efficacy (SE)	0.732	0.597	0.507			
Short video addiction (SVA)	0.809	0.796	0.691	0.725		
Short video flow (SVF)	0.749	0.663	0.441	0.792	0.739	

SVF = Short Video Flow, PA = Platform Attachment, PI = Peer Influence, SVA = Short Video Addiction, LF = Lying Flat, SE = Self-Efficacy.

### 4.3 Assessment of structural model

**Figure 2:**
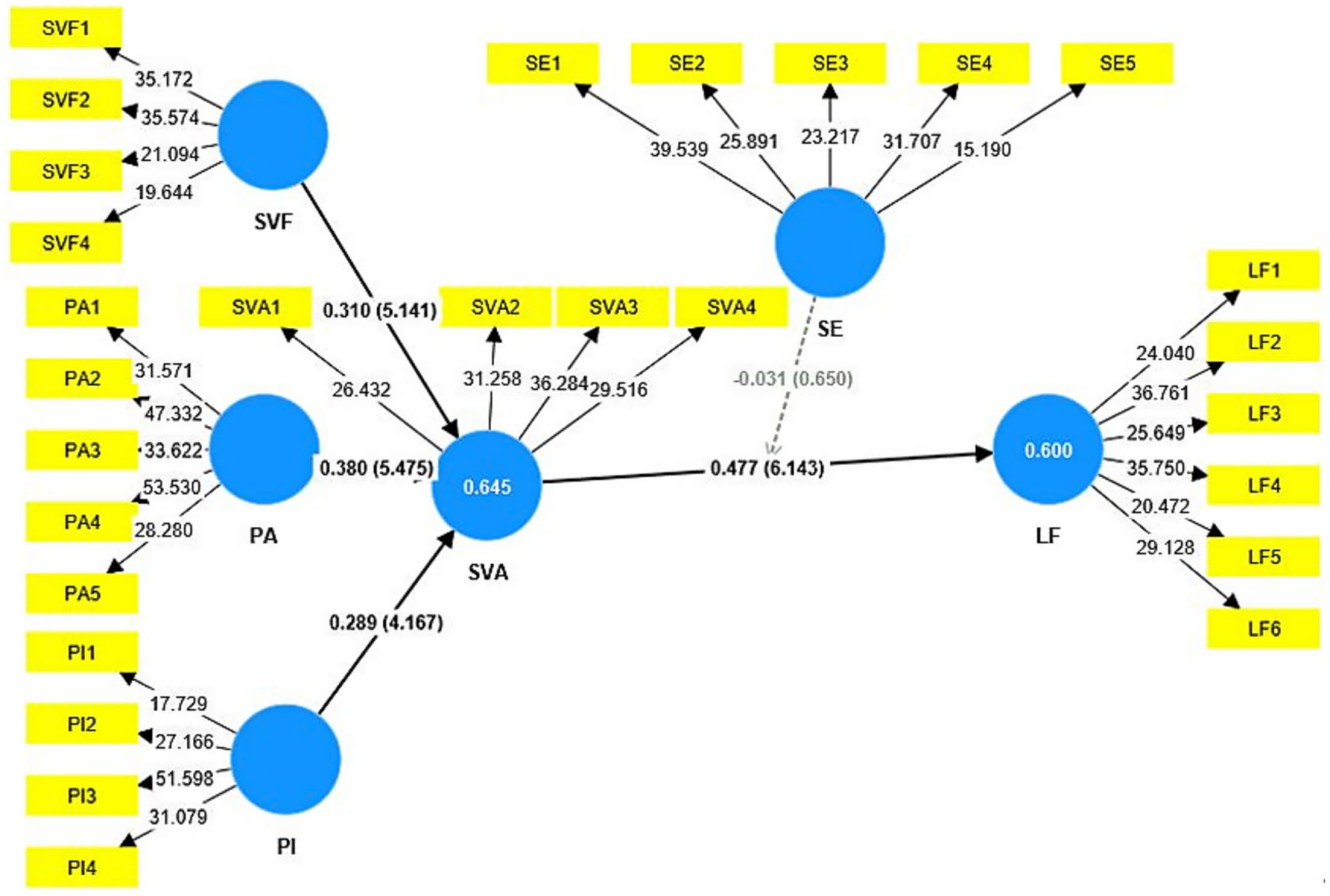
PLS-SEM statistical model diagram. SVF = Short Video Flow, PA = Platform Attachment, PI = Peer Influence, SVA = Short Video Addiction, LF = Lying Flat, SE = Self-Efficacy.

Following the suggestions of Becker et al. (2023), we reported the path coefficients, the standard errors, *t-*values and *p*-values for the structural model using a 10,000-sample re-sample bootstrapping procedure. Table 4 illustrates the direct relationships between the latent variables with which the present study is concerned. In addition, PLS results are shown in Figure 2. The result shows that SV flow (*β* = 0.310, *p* < 0.001, *f*^2^ = 0.175), platform attachment (*β* = 0.380, *p* < 0.001, *f*^2^ = 0.229), and peer influence (*β* = 0.289, *p* < 0.001, *f*^2^ = 0.170) are positively associated with SV addiction, thereby confirming H1, H2, and H3. Moreover, SV addiction (*β* = 0.477, *p* < 0.001, *f*^2^ = 0.298) is positively associated with lying flat, thus H4 is also supported.

**Table 4:** Hypothesis testing.

Hypothesis	Relationship	Std. beta	Std. dev	*t*-values	*p*-values	PCI LL	PCI UL	*f* ^2^	Result
H1	SVF➔ SVA	0.310	0.060	5.141	*p* < 0.001	0.209	0.408	0.175	supported
H2	PA➔ SVA	0.380	0.069	5.475	*p* < 0.001	0.269	0.498	0.229	supported
H3	PI➔ SVA	0.289	0.069	4.167	*p* < 0.001	0.174	0.404	0.170	supported
H4	SVA➔ LF	0.477	0.078	6.143	*p* < 0.001	0.344	0.599	0.298	supported

We use 90% confidence interval with a bootstrapping of 5,000. SVF = Short Video Flow, PA = Platform Attachment, PI = Peer Influence, SVA = Short Video Addiction, LF = Lying Flat, SE = Self-Efficacy.

The coefficient of determination (R2) quantifies the variance in endogenous variables explained by independent variable constructs and is the primary metric for assessing a model’s predictive capabilities. *R*^2^ values of 0.75, 0.50, and 0.25 are, respectively, considered to represent substantial, moderate, and weak determinants of variance (Hair et al., 2022). Based on Figure 2, in this study, *R*^2^ values for dependent variables, such as R2SVA = 0.645, and R^2^LF = 0.600, indicate that all *R*^2^ values meet the required standards for explanatory power. Moreover, we employed Stone-Geisse’s *Q*^2^ values to evaluate the predictive relevance of our model. The *Q*^2^ values for SVA and LF were 0.447, and 0.411, respectively, demonstrating substantial predictive relevance in accordance with the research objectives.

### 4.4 Testing the mediation effect

We used a bootstrapping method to examine the mediation effects of SV addiction. Table 5 shows that a significant association between SV flow (*β* = 0.148, *p* < 0.001, 95% CI [0.097, 0.198]), platform attachment (*β* = 0.181, *p* < 0.001, 95% CI [0.110, 0.265]), and peer influence (*β* = 0.138, *p* < 0.05, 95% CI [0.072, 0.212]) with lying flat through SV addiction, consistent with H5, H6, and H7.

**Table 5:** Mediation and moderation testing.

Hypothesis	Relationship	Std. beta	Std. dev	*t*-values	*p*-values	PCI LL	PCI UL	Result
H5	SVF➔ SVA➔ LF	0.148	0.031	4.791	*p* < 0.001	0.097	0.198	Supported
H6	PA➔ SVA➔ LF	0.181	0.047	3.844	*p* < 0 0.001	0.110	0.265	Supported
H7	PI➔ SVA➔ LF	0.138	0.043	3.205	*p* < 0.05	0.072	0.212	Supported
H8	SE*SVA ➔ LF	−0.031	0.048	0.650	*p* = 0.258	−0.111	0.048	Not supported

### 4.5 Testing the moderation effect

The moderating effect of SE on the relationship between SVA and LF was assessed using the two-stage latent interaction approach in SmartPLS 4.0. Bootstrapping with 5,000 resamples was employed to assess the significance of the interaction effect. As illustrated in Table 5, the interaction term between SE and SVA does not significantly predict LF (*β* = −0.031, *p* = 0.258, 95% CI [−0.111, 0.048]), indicating that the moderating effect (H8) is not statistically supported.

## 5 Discussion

### 5.1 The relationship between university students’ SV flow experience, platform attachment, peer effect, and SV addiction

According to the results of this study, SV flow experience is positive related to SV addiction, indicating that the flow experience of university students while watching SVs is a critical driving factor in their addiction. This finding is consistent with previous studies showing that flow states are positively related to addictive short video use across different populations, including adolescents and vocational school students (Lu et al., 2022; Nong et al., 2023; Ye et al., 2025a; Ye et al., 2025b). However, this study extends these findings by focusing on university students, whose greater autonomy and complex psychosocial needs may amplify the influence of flow experience on addictive behaviors. The results further support the notion that flow experience enhances dependence on SV platforms by intensifying feelings of immersion and enjoyment.

From the perspective of Social Cognitive Theory (SCT), this finding elucidates the central role of flow experience in SV addiction among university students, reinforcing the theoretical framework of the interaction between environment, behavior, and individual cognition (Qin et al., 2022). The immersive flow states created during SV use reinforce feelings of enjoyment and distort users’ sense of time, prompting increased use frequency and exacerbating addictive tendencies. These findings highlight that flow experience is not only a psychological state but also a behavior shaped by environmental factors, suggesting that university students’ addictive behaviors may arise from their heightened sensitivity to immersive experiences and a reliance on instant gratification.

Moreover, this study confirmed that Platform Attachment has a significant association with SV Addiction. The influence of platform attachment on addictive behavior has been validated in numerous studies. For example, Zhang et al. (2019) in their research on users’ attachment to SV platforms, found that high platform attachment significantly increases the likelihood of SV addiction. In addition, research on social media use has shown that individuals’ attachment-related tendencies are positively associated with problematic engagement with online platforms (D’Arienzo et al., 2019). However, Thadani and Cheung (2011) found that while platform attachment is positively associated with dependence behaviors in social network use, this effect may differ across different user groups and use scenarios. This highlights the need to consider the specific psychological and social backgrounds of the university student population when interpreting the influence of platform attachment on SV addiction.

Furthermore, this study found that Peer Influences is positively related to SV Addiction, which is aligned with previous studies. For instance, Ye et al. (2022) found that peer influence can significantly enhance adolescents’ tendency towards internet addiction when facing cyber victimization. This aligns with our finding that peer influences are significantly related to SV addiction, indicating that peers’ behaviors and attitudes while using SV platforms can greatly affect university students’ use behaviors. Similarly, Xu et al. (2023) found that peer pressure significantly predicted mobile social media addiction, indicating that peer-related social norms can drive higher levels of problematic engagement with online platforms. From the perspective of Social Cognitive Theory, peer influence shapes students’ engagement with SVs through environmental reinforcement and cognitive interaction, which may gradually strengthen dependency over time. Overall, these findings are consistent with our theoretical expectations, highlighting that peer influence is a key social factor contributing to SV addiction among university students.

### 5.2 The relationship between university students’ SV addiction and “lying flat” tendency

The results of this study confirmed that SV Addiction has a positive association with “Lying Flat” tendency. This indicates that the higher the level of SV addiction, the more likely university students are to adopt a “lying flat” attitude towards life. Many previous studies have pointed out that excessive use of SVs or other smartphone and social media platforms can lead to academic procrastination, lower academic achievement, and reduced attention spans in students (Ye et al., 2024a; Ye et al., 2024b; Ye et al., 2023a; Ye et al., 2023b; Chen et al., 2023; Nong et al., 2023). The rapid and short nature of SVs allows users to gain entertainment and satisfaction in a very short amount of time. This instant gratification weakens their commitment to long-term goals and plans. The immediate sense of satisfaction diminishes users’ interest and motivation in activities that require long-term investment, such as studying or working, thus leading them to adopt a “lying flat” mindset (Ye et al., 2022). In summary, SV addiction undermines university students’ commitment to long-term goals and may cause them to become disillusioned with real-life challenges and responsibilities, ultimately leading them to adopt a “lying flat” attitude toward life.

### 5.3 The mediating role of SV addiction between flow experience, platform attachment, peer influence, and lying flat

This study confirmed that SV Addiction acts as a significant mediator linking between flow experience, platform attachment, and peer influence on university students’ attitudes and behaviors, ultimately fostering the adoption of a “lying flat” lifestyle. This finding underscores the strong association between SV use and students’ attitudes toward life. The results support the proposed model, indicating that students’ cognitive, emotional, and social experiences indirectly shape their life attitudes through underlying psychological mechanisms, consistent with Social Cognitive Theory (Bandura, 2023). Flow experience, through its provision of immediate gratification and an avenue for escaping reality, reinforces students’ dependency on SVs. This phenomenon aligns with the findings of Qin et al. (2022), who noted that short-term satisfaction diminishes the pursuit of long-term goals, highlighting the complex psychological effects of SV addiction on university students. Meanwhile, platform attachment provides an emotional and behavioral foundation for this dependency, significantly increasing students’ engagement and frequency of use while fostering a tendency to avoid real-world challenges, echoing the results of Zhang et al. (2019). Furthermore, peer influence exacerbates this process through mechanisms such as imitation and social comparison as students tend to mirror peers’ media behaviors and compare their online engagement, which intensifies SV addiction and, in turn, reinforces their disengagement from real-world pressures. This pattern is consistent with previous studies showing that social comparison and peer validation significantly contribute to compulsive social media use and withdrawal-oriented behaviors (Feng et al., 2025; Zhang et al., 2025). These findings further highlight that SV addiction is not only a product of the interaction between external environmental factors and individual cognition but also plays a critical role in students’ avoidance of social competition and real-life pressures. This mediating mechanism underscores the importance of understanding addictive media use not only as a behavioral outcome but as a critical pathway influencing broader lifestyle choices among young adults. In this sense, the findings extend Social Cognitive Theory by illustrating how digital media environments shape life attitudes through behavioral and psychological mechanisms, offering a theoretical basis for understanding the “lying flat” phenomenon and guiding interventions for healthier media engagement.

### 5.4 The moderating role of self-efficacy

In this study, the moderating effect of Self-Efficacy was proposed as a key hypothesis, but the results showed that the moderating effect was not significant. Specifically, the analysis revealed that the interaction between self-efficacy and SV addiction did not have a significant impact on “lying flat” tendency, indicating that self-efficacy did not effectively moderate the relationship between SV addiction and “lying flat” attitude. This finding differs from some previous research. For example, Lin et al. (2023) found that high self-efficacy can mitigate the negative effects of SV addiction on creative self-efficacy and career interest, suggesting that self-efficacy generally buffers the negative consequences of addictive behaviors to some extent. Similarly, Zhou et al. (2022) indicated that academic self-efficacy played a moderating role between professional attitudes and academic burnout, with higher self-efficacy enabling students to better resist the pressures of addictive behaviors, thereby reducing the occurrence of academic burnout. However, this study did not validate such a moderating effect, which may reflect contextual or psychological differences in how self-efficacy functions across domains. Unlike prior research focusing on task-related or academic contexts, this study examined lifestyle disengagement behaviors such as “lying flat,” which may involve broader existential and motivational factors beyond individual self-belief. When external social and economic pressures are perceived as uncontrollable, even individuals with strong self-efficacy might experience reduced motivation to resist passive attitudes. This interpretation aligns with Social Cognitive Theory (Bandura, 2023), which posits that the influence of self-efficacy is conditional upon perceived environmental controllability.

## 6 Research implications

### 6.1 Academic contributions

This study explored the relationship between SV addiction and “lying flat” tendency among university students, specifically examining the roles of variables such as SV flow experience, platform attachment, peer influence, and self-efficacy. It provides the following academic contributions to the understanding of modern university students’ lifestyle and psychological motivations.

First, this study, grounded in Social Cognitive Theory (SCT), investigates how SV addiction influences “lying flat” tendency among university students through the combined effects of environmental factors (platform attachment, peer influence), cognitive processes (flow experience), and personal characteristics (self-efficacy). By further enriching the application of SCT in the context of digital media behaviors, this research broadens the theoretical understanding of behavioral mechanisms in the digital environment. Moreover, the study examines the mediating role of SV addiction and its association with individuals’ life attitudes, expanding SCT’s applicability in explaining university students’ lifestyle choices and providing theoretical support for future research on behavioral patterns in digital environments. Furthermore, this study extends SCT by focusing on the role of personal characteristics, such as self-efficacy, in shaping the pathways through which digital addiction affects behavior, highlighting the importance of individual differences in digital media use. By exploring the moderating effect of self-efficacy between SV addiction and “lying flat” tendency, this research enriches the understanding of how individual factors contribute to behavioral patterns. Although the moderation effect was not statistically significant, this exploration offers new insights into the variability of digital addiction behaviors among individuals.

Second, the current research systematically reveals the link between SV addiction and university students’ lifestyles, focusing on how digital media use shapes individuals’ life attitudes and choices through behavioral pathways. SV addiction, as a critical variable, reflects the combined effects of SV flow experience, platform attachment, and peer influence in the formation of addictive behaviors, while also highlighting the far-reaching implications of digital media use on students’ psychological states and social participation. Specifically, SV addiction reinforces instant gratification and virtual accomplishment, weakening students’ pursuit of real-life goals. Moreover, excessive reliance on digital media reduces social connectedness and a sense of achievement in real life. Building upon existing literature, this study further expands the understanding of SV addiction by demonstrating that this behavior not only affects individuals’ short-term psychological states but also alters their long-term behavioral patterns and life attitudes through mediating mechanisms.

Additionally, while existing studies have extensively examined the relationships between internet addiction and variables such as mental health and academic performance, few have focused on how SV addiction influences university students’ life attitudes and “lying flat” tendency. This study fills this research gap, deepens the understanding of the psychological and behavioral pattern associated with digital media use among university students, and provides a novel theoretical framework and academic perspective for exploring the relationship between digital media and the lifestyles of young people.

Third, this study reveals the critical role of SV addiction in shaping the “lying flat” tendency, demonstrating that excessive use of SVs weakens university students’ motivation to study and pursue life goals, leading them to adopt “lying flat” as a strategy to cope with stress and challenges. Chen et al. (2023) found that SV addiction often results in issues such as reduced attention span and diminished learning motivation, which may prompt individuals to avoid real-life responsibilities and adopt a “lying flat” lifestyle. This study further validates the link between SV addiction and negative behaviors among university students and expands the understanding of the “lying flat” phenomenon. A unique contribution of this research lies in its comprehensive analysis of the multifaceted psychological drivers behind the “lying flat” phenomenon, incorporating factors such as digital media use, social comparison, and cognitive processes. This multi-level analysis not only provides a deeper understanding of how SV addiction undermines the pursuit of real-life goals through mechanisms like instant gratification and avoidance tendencies but also clarifies the intrinsic pathways linking SV use to “lying flat” tendency.

### 6.2 Practical contributions

This study provides several important insights and recommendations for educational administrators, policymakers, and mental health professionals to address the growing issues of “lying flat” tendency and SV addiction among university students.

Firstly, this study finds that SV addiction has a significant positive effect on “lying flat” tendency among university students. Education administrators and mental health professionals should prioritize addressing the detrimental effects of SV addiction on university students’ mental health and attitudes toward life by implementing targeted interventions to mitigate addictive behaviors. For instance, self-efficacy enhancement programs combined with self-regulation training could be incorporated into mental health courses. Cognitive-behavioral therapy can be employed to help students strengthen self-control and reduce excessive reliance on SVs, thereby lowering the likelihood of “lying flat” tendency (Huang et al., 2021). Additionally, mental health interventions should emphasize fostering students’ sense of realistic goals and encouraging a proactive attitude toward challenges to help them balance their engagement in both virtual and real-life spheres.

Secondly, this study highlights the critical role of peer influence in SV addiction and “lying flat” tendency, underscoring the need for educators to provide positive guidance in university students’ daily lives. Research has shown that peer groups have a significant influence on individual behavior (Poldin et al., 2016). Educators can promote classroom discussions and extracurricular collaborative activities to enhance students’ sense of group belonging and reinforce positive behaviors. Such efforts can guide students toward achieving a healthy balance between digital media use and the pursuit of real-world goals.

## 7 Research limitations and future directions

This study is based on a sample of Chinese university students, which may limit the generalizability of the findings. Given potential differences in cultural, socioeconomic, and educational contexts, variations in SV addiction and “lying flat” tendencies may occur, and findings should be interpreted with caution in other populations. Additionally, this study employed a cross-sectional design, which restricts the ability to infer the temporal or causal relationships among variables. Future research could adopt longitudinal or experimental designs to further validate the directionality of these relationships. Moreover, the analysis primarily focuses on the associations between SV addiction, flow experience, platform attachment, peer influence, and the tendency to “lie flat,” but does not account for other potentially relevant psychosocial, educational, or technological variables, such as personality traits, family environment, socioeconomic status, educational background, or platform-level features like recommendation algorithms. Some of these factors may serve as confounders, particularly in the relationship between short video addiction and the “lying flat” mindset. It is worth noting that SE, although examined in this study as a moderator, may also serve as an antecedent of SVA, thereby indirectly influencing the “lying flat” tendency. Therefore, future research could incorporate a wider range of variables to gain a more comprehensive understanding of the causes behind the “lying flat” phenomenon.

## 8 Conclusion

This study investigated the psychological mechanisms underlying the “lying flat” tendency among Chinese university students within the framework of social cognitive theory. The findings revealed that SV addiction served as a central mediating mechanism linking flow experience, platform attachment, and peer influence to the “lying flat” tendency. Higher levels of SV addiction were associated with stronger disengagement and a passive lifestyle orientation, underscoring a close association between addictive media use and challenges to youth well-being. Although flow experience, platform attachment, and peer influence predicted SV addiction, this study highlights that the critical pathway lies in how addiction itself contributes to the emergence of the “lying flat” mindset. In contrast, the moderating role of self-efficacy was not significant, indicating that its buffering effect may be limited in the context of excessive media engagement. This study contributes to the literature by extending social cognitive theory to the digital media context and addressing the psychological mechanisms through which SV addiction fosters lifestyle disengagement among university students. Universities should prioritize fostering students’ self-regulation and digital literacy to mitigate excessive media engagement and prevent maladaptive coping tendencies such as “lying flat.” Cultivating a sense of realistic goals and life purpose, together with strengthening peer support and positive social interactions, may further help students achieve a healthy balance between virtual engagement and real-life participation.

## Data Availability

The raw data supporting the conclusions of this article will be made available by the authors, without undue reservation.

## Appendix: questionnaire

**Table:**
